# Complete mitochondrial genome of the wild *Diptychus maculatus* (Cypriniformes, Cyprinidae, Schizothoracinae) from Yeken River using next generation sequencing and the phylogenetic relationship of Cyprinidae species

**DOI:** 10.1080/23802359.2020.1715288

**Published:** 2020-01-20

**Authors:** Jiangong Niu, Yu Zhang, Hong Liu, Jiangwei Hu, Lingang Cai, Renming Zhang, Hui Zhang

**Affiliations:** a Xinjiang Fishery Research Institute, Urumqi, China;; b Laboratory for Marine Ecology and Environmental Science, Qingdao National Laboratory for Marine Science and Technology, Qingdao, China;; c CAS Key Laboratory of Marine Ecology and Environmental Sciences, Institute of Oceanology, Chinese Academy of Sciences, Qingdao, China;; d Center for Ocean Mega-Science, Chinese Academy of Sciences, Qingdao, China

**Keywords:** *Diptychus maculatus*, wild fish, mitochondrial genome, next generation sequencing

## Abstract

The complete mitochondrial genome of the wild *Diptychus maculatus* collected from Yeken River was determined using next generation sequencing. The mitogenome is a circular molecule 16,765 bp in length, including 13 protein-coding genes, two ribosomal RNA genes, 22 transfer RNA genes, and a control region. The TAS, central CSB, and CSB were detected in the control region. The gene contents of the mitogenome are identical to those observed in most bony fishes. The NJ phylogenetic tree showed that *D. maculatus* clustered into one separate branch which is close to genus *Gymnodiptychus* from the same subfamily.

Next generation sequencing (NGS) has revolutionized the field of molecular biology through the rapid and cost effective collection of large amounts of genomic data (Schuster 2008). NGS could provide an effective platform for the development of the mitochondrial genome that can be used to provide insight into population processes and the evolutionary history of species. By exploiting certain tissue types, such as muscle, total genomic DNA extractions can contain high concentrations of mitochondrial DNA which may then be overrepresented in NGS analyses (Dalziel et al. 2005).

*Diptychus maculatus* mainly distributes in the Tarim River Basin and the Ili River-Balkhash Lake Basin, and it is one of the Class II protected fishes in Xinjiang China (Niu et al. 2015). However, there are few studies on its wild population from genome aspects. In the present study, we use NGS using Illumina Hiseq analysis to determine the mitogenome of the wild *D. maculatus* collected from Yeken River (37°49′47.55″N, 75°31′3.95″E) in May 2019. The specimen is preserved in the fish herbarium of Xinjiang Fishery Research Institute with the No. BAN-2019-05-02.

The complete mitogenome of *D. maculatus* was 16,765 bp in length (GenBank accession no. MN413609), with the nucleotide composition as A (27.65%), T (26.75%), G (19.08%), and C (26.53%). As in other vertebrates (Miya et al. 2001), it contained 13 protein-coding genes, two rRNA genes (12S rRNA and 16S rRNA), 22 tRNA genes, and a control region. Most mitochondrial genes of *D. maculatus* were encoded on the H-strand, with only ND6 and eight tRNA (Gln, Ala, Asn, Cys, Tyr, Ser-UCN, Glu, and Pro) genes encoded on the L-strand. Among 13 protein-coding genes, two overlapping reading frames were detected on the same strand. The ATPase 6 and ATPase 8 overlap by seven nucleotides, and ND4 and ND4L share seven nucleotides. ND5 and ND6 overlap by four nucleotides on the opposite strand. ATG is the initiation codon of all protein-coding genes. TAA is the stop codon for six genes (ND6, COI, ATPase 6, COIII, ND4L, and ND5), the other genes have incomplete stop codons TA– or T––, which are presumably completed as TAA by post-transcriptional polyadenylation (Ojala et al. 1981). The 12S and 16S ribosomal RNA genes of *D. maculatus* comprise 953 bp and 1637 bp, respectively. They are located between tRNA^Phe^ and tRNA^Leu^ (UUR) as they are in other vertebrates (Zhang and Xian 2018). The 22 tRNA genes are interspersed in the genome and range in size from 64 to 75 bp and fold into cloverleaf secondary structures with normal base paring. The control region of *D. maculatus* is located between tRNA^Pro^ and tRNA^Phe^ and was determined to be 815 bp in length. The TAS, central CSB, and CSB were detected in the control region, which is similar to most bony fishes (Zhang et al. 2013). Phylogenetic relationship was revealed by NJ tree among 16 Cyprinidae species based on complete mitogenome. The NJ phylogenetic tree showed that *D. maculatus* clustered into one separate branch which is close to genus *Gymnodiptychus* from the same subfamily (Figure 1).

**Figure 1.**
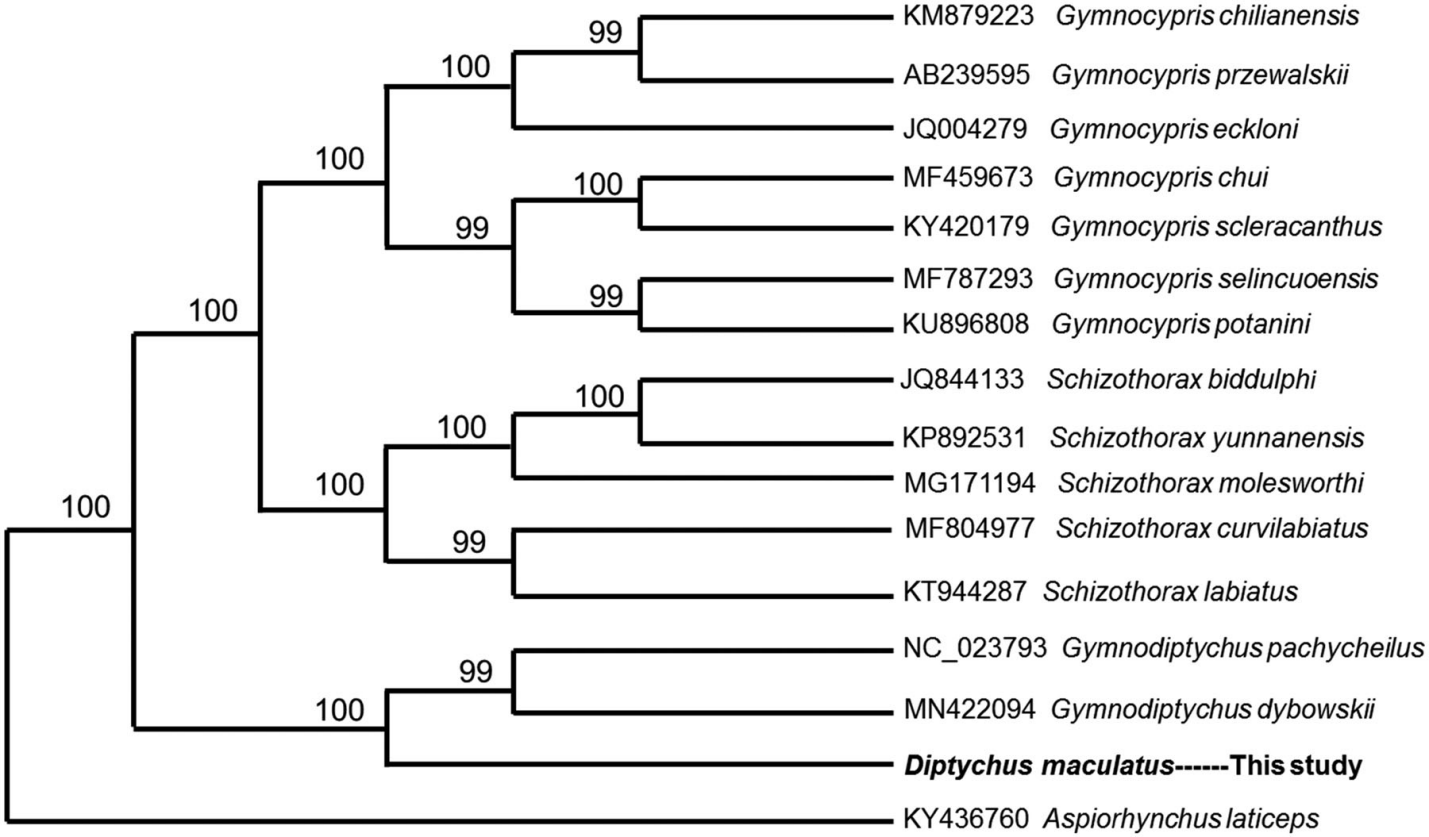
Phylogenetic relationship revealed by NJ tree among 16 Cyprinidae species.
